# Global burden of bladder cancer mortality in 2020 and 2040 according to GLOBOCAN estimates

**DOI:** 10.1007/s00345-024-04949-8

**Published:** 2024-04-16

**Authors:** András Wéber, Jerome Vignat, Richa Shah, Eileen Morgan, Mathieu Laversanne, Péter Nagy, István Kenessey, Ariana Znaor

**Affiliations:** 1 Hungarian National Cancer Registry and National Tumor Biology Laboratory, National Institute of Oncology, Budapest, Hungary; 2 Cancer Surveillance Branch, International Agency for Research on Cancer, Lyon, France; 3 Department of Molecular Immunology and Toxicology and the National Tumor Biology Laboratory, National Institute of Oncology, Budapest, Hungary; 4 Department of Anatomy and Histology, HUN-REN–UVMB Laboratory of Redox Biology Research Group, University of Veterinary Medicine, Budapest, Hungary; 5 Chemistry Institute, University of Debrecen, Debrecen, Hungary; 6 Department of Pathology, Forensic and Insurance Medicine, Semmelweis University, Budapest, Hungary

**Keywords:** Bladder cancer, Mortality, Projection

## Abstract

**Introduction:**

In 2020, bladder cancer (BC) was the seventh most prevalent cancer in the world, with 5-year prevalence of more than 1.7 million cases. Due to the main risk factors—smoking and chemical exposures—associated with BC, it is considered a largely preventable and avoidable cancer. An overview of BC mortality can allow an insight not only into the prevalence of global risk factors, but also into the varying efficiency of healthcare systems worldwide. For this purpose, this study analyzes the national mortality estimates for 2020 and projected future trends up to 2040.

**Materials and methods:**

Age-standardized mortality rates per 100,000 person-years of BC for 185 countries by sex were obtained from the GLOBOCAN 2020 database, operated by the International Agency for Research on Cancer (IARC). Mortality rates were stratified according to sex and Human Development Index (HDI). BC deaths were projected up to 2040 on the basis of demographic changes, alongside different scenarios of annually increasing, stable or decreasing mortality rates from the baseline year of 2020.

**Results:**

In 2020, nearly three times more men died from BC than women, with more than 210,000 deaths in both sexes combined, worldwide. Regardless of gender, more than half of the total BC deaths were from countries with a very high HDI. According to our projections, while the number of deaths for men can only increase up to 54% (from 159 to around 163–245 thousand), for women it is projected to increase two- to three-fold (from 50 to around 119–176 thousand) by 2040. The burden of BC mortality in countries with a very high HDI versus high HDI appears to converge by 2040 for both sexes.

**Conclusion:**

Opposite mortality trends by gender highlight the urgent need for immediate interventions to expand anti-tobacco strategies, especially for women. The implementation of more strict occupational health and safety regulations could also prevent exposures associated with BC. Improving the ability to detect BC earlier and access to treatment can have a significant positive impact on reducing mortality rates, minimizing economic costs, and enhancing the quality of life for patients.

## Introduction

In 2020, over 573 thousand new bladder cancer (BC) cases and around 212 thousand BC deaths were estimated to occur worldwide [1], representing approximately 3% of all new cancer cases and more than 2% of cancer deaths. BC ranks as the tenth most common cancer diagnosed, and the 13th most common cause of cancer mortality, worldwide and it is effectively treatable when diagnosed early with relatively high survival with 5-year relative survival rates of around 77%observed in the US [2] and 68% reported in Europe [3].

The main risk factor for BC is tobacco smoking; male smokers have a 3.3, women smokers have a 2.2 times higher risk of BC compared to nonsmokers [4], and thus is a highly avoidable cancer. Additionally, experience derived from the global smoking epidemic can be applied to obtain a better understanding of the background trends, gender differences and possible future scenarios of BC, especially in higher income settings [5, 6]. At the same time, occupational and environmental toxins may also contribute to the development of this disease [7].

This study aims to describe the burden of BC mortality across different world regions in 2020 based on GLOBOCAN estimates from the International Agency for Research on Cancer (IARC). Our paper also examines the gender-based BC mortality distribution in terms of geographic variation and Human Development Index (HDI), as well as projecting the future burden up to 2040. These disparities can express differences in healthcare systems, treatment protocols, or varying levels of access to diagnosis and treatment facilities, particularly between countries at different levels of development. Understanding these patterns can inform public health experts on ways of reducing the global burden of BC and improving health outcomes.

## Materials and methods

The number of deaths from cancers of the bladder, International Classification of Diseases, 10th revision (ICD-10): C67 was extracted from the GLOBOCAN 2020 database for 185 countries or territories, by sex and 18 age groups (0–4, 5–9, 10–14…75–79, 80–84, 85, and over) [8–10]. Corresponding population data for 2020 were extracted from the United Nations (UN) website [11]. The data sources and hierarchy of methods used in compiling the cancer estimates have been described in detail elsewhere [9]. In brief, the GLOBOCAN estimates are assembled at the national level using the best available sources of cancer incidence and mortality data within a given country. The methods used to derive the 2020 estimates correspond to those used for previous years [12–14]; where applicable, priority is given to short-term predictions and modeled mortality to incidence (M:I) ratios, while validity is dependent on the degree of representativeness and quality of the source information [9].

We present tables and figures based on the estimated number of deaths, as well as two summary measures using direct standardization, namely, the age-standardized mortality rate (ASR) per 100,000 person-years based on the 1966 Segi–Doll World standard population [15, 16], and the cumulative risk of developing or dying from cancer before the age of 75 years expressed as a percentage, assuming the absence of competing causes of death [17]. These measures allow comparisons between populations adjusted for differences in age structures. We also provide a prediction of the future number of BC deaths worldwide for the year 2040 applying uniformly increasing (+ 3%, + 2%, + 1%), stable (0%), or decreasing (− 1%, − 2%, − 3%) mortality rates from the baseline year of 2020 to the estimations of UN population data.

The possible impact of the COVID-19 pandemic was not taken into consideration in the calculations. The results are presented by country, and aggregated, across 20 UN-defined world regions [11], and according to the UN’s four-tier Human Development Index (HDI) in 2020 [18], the latter being a means to assess the cancer burden at varying levels of development (low, medium, high and very high HDI). Throughout we use the terms transitioning, emerging and lower HDI countries/economies as synonyms for nations classified as low or medium HDI, and transitioned or higher HDI countries/economies for those classified as high or very high HDI.

The Global Cancer Observatory (GCO, https://gco.iarc.fr) includes facilities for the tabulation and graphical visualization of the GLOBOCAN database, including explorations of the current [8] and future [19] burden for 36 cancer types, and all cancers combined, including non-melanoma skin cancer (ICD-10 C44 excluding basal cell carcinomas).

## Results

### Bladder cancer mortality—national rankings 2020

According to the GLOBOCAN estimates for 2020, nearly three times more men died due to BC than women worldwide, with more than 210 thousand deaths combined (Table 1).

**Table 1:** Distribution of population and bladder cancer mortality (deaths, rates, risks) by world region in 2020, by gender and HDI level

	Male						Female					
	Population (in millions)		Deaths				Population (in millions)		Deaths			
	*N*	% in world total	*N*	% in world total	ASR (world)	Cum. risk	*N*	% in world total	*N*	%	ASR (world)	Cum. risk
Eastern Asia	844,784	21.4	39,717	25.0	2.7	1.4	818,396	21.0	14,489	27.0	0.8	0.4
All but China	116,790	3.0	10,039	6.3	2.7	1.5	121,460	3.1	4774	8.9	0.9	0.4
China	727,994	18.5	29,678	18.7	2.7	1.3	696,936	17.9	9715	18.1	0.7	0.4
Western Europe	95,684	2.4	15,341	9.7	5.1	2.6	99,374	2.5	5525	10.3	1.4	0.6
Northern America	185,394	4.7	15,129	9.5	3.5	1.8	188,562	4.8	5916	11.0	1.1	0.5
South-Central Asia	1,044,801	26.5	14,838	9.3	1.7	0.6	999,541	25.6	3405	6.3	0.4	0.1
All but India	323,804	8.2	5893	3.7	2.0	0.7	324,151	8.3	1196	2.2	0.5	0.1
India	720,997	18.3	8945	5.6	1.4	0.5	675,390	17.3	2209	4.1	0.3	0.1
Eastern Europe	137,792	3.5	14,797	9.3	6.0	2.4	155,042	4.0	4132	7.7	0.9	0.4
Southern Europe	74,394	1.9	14,000	8.8	5.9	2.9	78,109	2.0	3931	7.3	1.2	0.6
Northern Africa	126,865	3.2	8769	5.5	9.2	4.0	124,553	3.2	2018	3.8	1.8	0.8
South-Eastern Asia	335,751	8.5	7857	4.9	2.6	1.2	334,414	8.6	2470	4.6	0.6	0.3
South America	213,015	5.4	7067	4.5	2.6	1.3	218,515	5.6	3041	5.7	0.8	0.4
Northern Europe	52,303	1.3	6678	4.2	4.3	2.3	53,528	1.4	2885	5.4	1.5	0.7
Western Asia	150,010	3.8	6472	4.1	5.9	2.6	136,628	3.5	1362	2.5	1.0	0.4
Eastern Africa	222,752	5.6	2298	1.4	2.6	1.1	226,541	5.8	1628	3.0	1.4	0.5
Western Africa	205,216	5.2	1386	0.9	1.5	0.5	202,909	5.2	1011	1.9	1.0	0.3
Central America	86,538	2.2	1207	0.8	1.3	0.6	89,806	2.3	483	0.9	0.4	0.2
Australia and New Zealand	15,246	0.4	1160	0.7	2.9	1.8	15,485	0.4	455	0.8	1.0	0.5
Caribbean	21,760	0.6	917	0.6	2.8	1.3	22,203	0.6	385	0.7	1.0	0.4
Southern Africa	32,737	0.8	566	0.4	2.7	1.1	34,535	0.9	287	0.5	0.9	0.3
Middle Africa	91,791	2.3	483	0.3	1.3	0.5	92,779	2.4	301	0.6	0.7	0.3
Melanesia	6154	0.2	85	0.1	2.7	0.9	5805	0.1	19	0.0	0.6	0.2
Polynesia	359	0.0	12	0.0	3.4	0.8	353	0.0	7	0.0	1.8	0.6
Micronesia	268	0.0	6	0.0	2.3	0.3	263	0.0	1	0.0	0.3	0.3
Very high HDI	619,171	15.7	80,336	50.6	4.4	2.2	624,936	16.0	28,377	52.8	1.1	0.5
High HDI	1,260,146	32.0	57,133	36.0	3.1	1.4	1,252,331	32.1	18,164	33.8	0.8	0.4
Medium HDI	1,700,996	43.1	16,414	10.3	1.7	0.6	1,655,051	42.5	4305	8.0	0.4	0.1
Low HDI	348,834	8.8	4812	3.0	2.3	0.9	350,353	9.0	2881	5.4	1.1	0.4
World	3,943,612	100.0	158,785	100.0	3.3	1.6	3,897,341	100.0	53,751	100.0	0.9	0.4

ASR = Age-standardized rate, Population standard: Segi–Doll’s world standard population, HDI = Human Development Index, Cum. Risk = risk of dying from cancer before the age of 75 expressed as a percentage, assuming the absence of competing causes of death

**Fig. 1:**
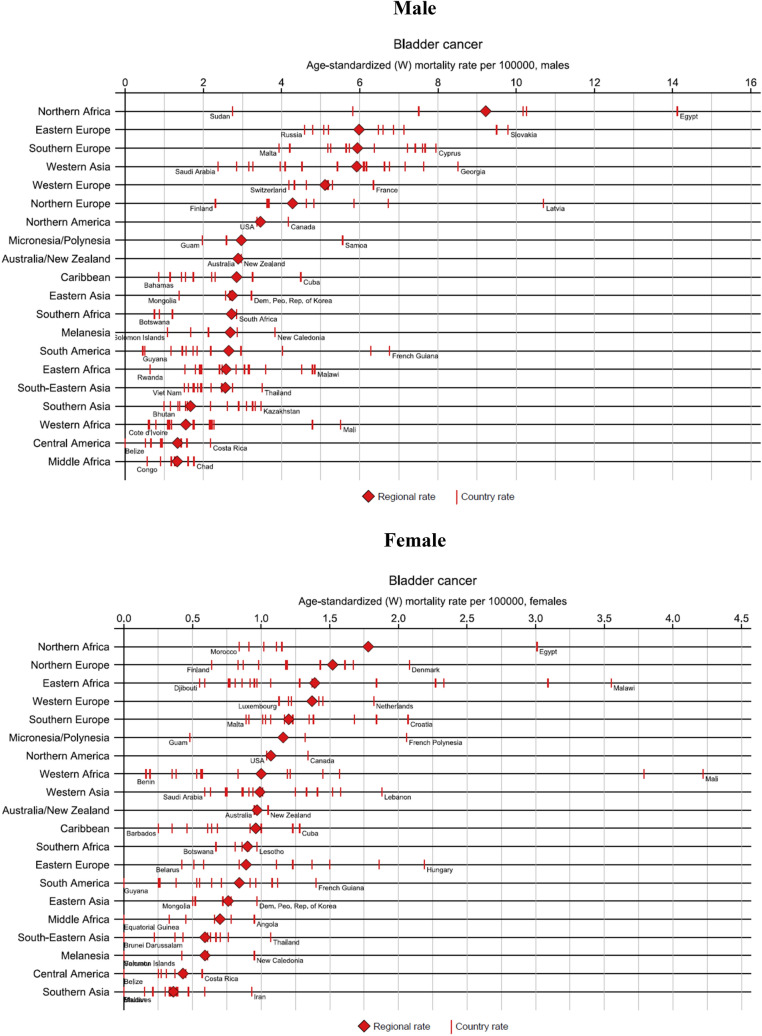
Bladder cancer age-standardized mortality rates per 100,000 by world regions and sex in 2020. Population standard: Segi–Doll’s world standard population

**Fig. 2:**
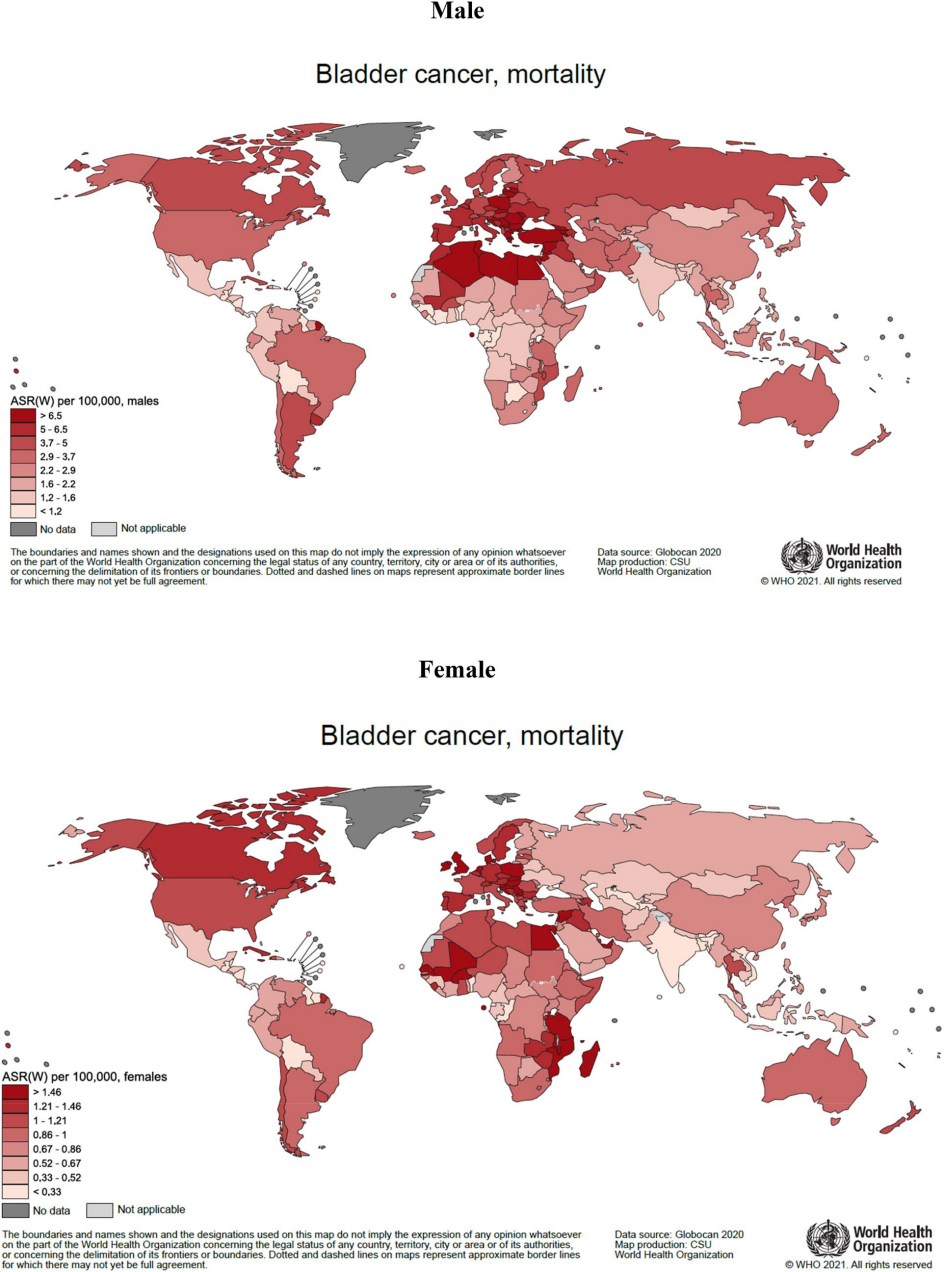
Bladder cancer mortality measured by age-standardized rates per 100,000 for the countries of the world in 2020. Population standard: Segi–Doll’s world standard population

Male (age-standardized) mortality rates are the highest in Northern Africa and Eastern Europe (especially Egypt, with rates of more than 14 per 100,000 and Slovakia, at nearly 10 per 100,000), Southern Europe (particularly Cyprus) and Western Asia, while rates are the lowest in Western and Middle Africa, and Central America (Figs. 1 and 2). Female mortality rates are the highest in Northern Africa, Northern Europe, Eastern Africa, and Western Europe, specifically Egypt, Denmark, Malawi, and the Netherlands, respectively. Somewhat lower rates are observed in South-Eastern Asia, Central America, and Southern Asia. Gender differences, are the greatest in Northern Africa, Eastern Europe, Western Asia, and Southern Europe, and the smallest differences observed in Central America and Middle and Western Africa.

### Bladder cancer mortality burden by 2040

**Fig. 3:**
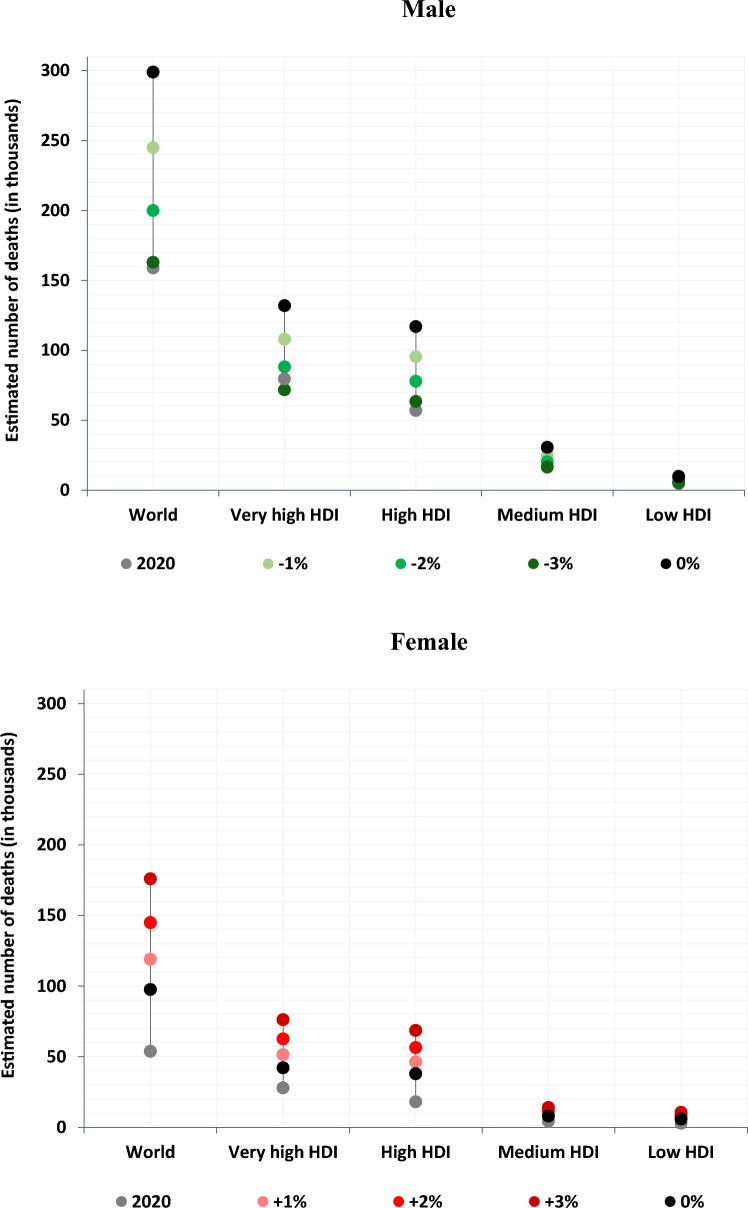
Estimated number of bladder cancer deaths from 2020 to 2040, using different scenarios on the change in annual mortality rates and levels on the Human Development Index (HDI)

If the current mortality rates were to remain constant over the next two decades i.e., assuming the 0% forecast scenario, nearly 300 thousand male BC deaths are predicted to occur in 2040, compared to 159 thousand in 2020 (Fig. 3). For women, the corresponding deaths are approximately a third of their male counterparts, yielding a predicted increase from 50,000 deaths in 2020 to around 100 thousand in 2040. The projection also considered varying rates of change between − 3% and + 3% per year for BC mortality. Considering the presumed scenarios of the smoking epidemic in the short-term future, a significant increase in BC mortality for women is to be expected, compared to a general decline in men’s figures, with national or regional exceptions. The projected number of BC deaths among men will likely range between 163 and 245 thousand (scenarios with decreasing rates of change at − 3%, − 2%, − 1% per year) and for women between 119 and 176 thousand (scenarios with increasing rates of change at 1%, 2%, 3% per year) by 2040. Under all scenarios, the burden of BC mortality in very high versus high HDI countries will converge noticeably for both sexes by 2040. The disease burden in medium- and low-HDI countries is expected to remain low.

## Discussion

Our study reports the current mortality status of BC worldwide and describes its increasing global burden up to 2040 by sex and HDI levels based on demographic projections.

Due to the association between smoking and BC, the global smoking epidemic serves as useful input to our understanding of the present and future epidemiology of BC. While smoking prevalence for men has been declining in high and very high-income settings, e.g., in Northern America, Europe and Australia, and New Zealand concerns remain among women in low-income countries (for instance mostly in Asian and African countries) where presently low levels of cigarette consumption are already on the rise. In an era where women’s spending power is increasing, cigarettes are becoming affordable and women are more exposed to the marketing strategies of tobacco companies, in an environment where cultural constraints are weakening and female-specific quitting programs are rare [20–22].

Women are typically diagnosed with more advanced cancer and have a worse prognosis than men [23]. Reasons for this may be anatomical differences, different time lags from first symptoms to diagnosis and variations in hormone receptors [24]. However, women were more likely to be treated for voiding complaints or possible urinary tract infections without further evaluation or referral to urology than men [25]. As a result, a higher proportion of women may appear in the healthcare system with more advanced stage and more extensive metastases [26]. Other researchers suggest that hormonal factors, such as menopausal status and age at menopause, alongside smoking, may play a role in modifying BC risk among women [27]. All of this contributes to a further closing of the gender gap in BC mortality in future for high and very high-income HDI settings: as reported by our study, while the number of deaths for men could only increase by about 50%, for women, it could double or triple by 2040, according to the most probable scenarios, calculated with decreasing annual percentages of mortality change: − 3%, − 2%, − 1% per year for men and increasing by + 1%, + 2%, + 3% per year for women.

Besides tobacco consumption, occupational or environmental toxins may also contribute greatly to the burden of this disease. A previous cohort study from Denmark investigated how air, sea and ground pollution caused by a chemical factory contributed to an increased cancer risk [28]. Exposure to aromatic amines and other chemicals affecting workers in the painting, rubber, or aluminum industries have been confirmed as risk factors of bladder cancer [29]. Moreover according to an EU-wide investigation, long-term exposure to disinfection by-products in drinking water (trihalomethanes) has been consistently associated with an increased risk of BC [30]. In summary, the main risk factor for BC is tobacco smoking, which is responsible for more than half of BC cases and occupational or environmental toxins contributing to approximately one-fifth of all cases [31]. However, the precise proportion can be difficult to estimate as BC develops decades after exposure, even if the exposure only lasted several years. Schistosomiasis infection is also a common cause of this disease triggering related squamous cell carcinoma in regions of Africa and the Middle East, which infers a poorer prognosis in comparison to urothelial carcinoma. With approximately four-fifth of cases attributable to known modifiable risk factors, BC can be considered as largely preventable and greatly benefit from targeted prevention strategies [31].

Differences in healthcare systems, treatment protocols, or varying levels of access to diagnosis and treatment facilities, particularly between countries at different levels of development, can also partly explain the differences observed in BC survival and mortality rates between countries [3, 32]. Furthermore, BC is one of the most challenging and expensive cancers to diagnose and treat: its diagnosis relies on cystoscopy, an invasive and expensive procedure that might not be easily accessible in low-resource settings [23, 33]. In light of these facts, one result of our research has particular importance: the decreasing difference of the BC mortality burden between very high versus high HDI countries. Reasons for this could include the aging population, changing behaviors in tobacco consumption, the modification of other risk factors and diversity in treatment availability, where generally, very high HDI countries have better regulations and enforcement of environmental standards than lower HDI countries. Further in-depth researches would be necessary to examine and reveal the exact role of these factors, because most of them (e.g., impact of smoking or environmental risk factors) were not applied in our prediction model. In addition, the modest changes indicated by our projections in medium and low-HDI countries may be misleading, because these can be influenced to a large degree by the changing exposure to tobacco consumption and environmental hazards.

Various social–political interventions might alleviate the global burden of BC in future. The end of smoking may be achieved with well-known anti-smoking strategies, such as raising the value-added tax (VAT), a gradual ban on cigarette use in the community, and effectively targeted anti-cigarette campaigns, can significantly decrease the risk of BC [31]. In order to achieve this, it would be particularly necessary to target women around the world, especially in middle- and low-income populations. Besides this, stricter occupational health, safety regulations and the right targeting of preventive interventions could also reduce exposures which can cause BC.

Improving the ability to detect, for example through improving access to cystoscopy, and monitor BC can have a significant positive impact on reducing mortality rates, minimizing economic costs, and enhancing the quality of life for patients. Women should be a target population, as they generally are more likely to receive a diagnosis at advanced stages and have a poor prognosis.

The limitations of this study include the wide degree of variability in local data recording practices and the quality of BC causes of death data from death registries i.e., low rates observed in some regions could reflect difficulties in ascertaining and certifying causes of death. For instance, most African and some Asian countries have weak mortality statistics systems. In GLOBOCAN, in countries where mortality series were not available from national vital registration sources, the predominant means of the estimation of rates were from corresponding national incidence estimates via modeling, using incidence-to-mortality ratios derived from cancer registries in neighboring countries. However, higher rates in other regions may be affected to an unknown extent by the proportion of autopsies performed among the deceased. Our prediction model does not consider the changing smoking prevalence in the past as a key determinant of present and future BCs. However, we provide possible scenarios on the basis of uniform increases or decreases in rates that may help provide a realistic overview of the changing future burden of BC.

## Conclusion

BC is a significant public health issue globally, with mortality rates showing geographical variation and higher rates among men than women at present. However, estimates indicate that mortality rates will increase for women in the future, with the burden of BC in high HDI countries converging to very high HDI countries for both men and women. Treatment protocols, healthcare systems, and access to diagnosis and treatment facilities can partly explain the differences observed in BC survival and mortality rates between different regions. This study recommends social and political interventions to alleviate the global burden of BC in future. It suggests smoking cessation according to well-known anti-smoking strategies and campaigns, and effective workplace safety practices. Improving the ability to detect, monitor and treat BC can have a significant positive impact on reducing mortality rates, minimizing economic costs, and enhancing the quality of life for patients.

## Data Availability

Data are available in a public, open access repository.
